# Responses of Salt Marsh Plant Rhizosphere Diazotroph Assemblages to Drought

**DOI:** 10.3390/microorganisms6010027

**Published:** 2018-03-15

**Authors:** Debra A. Davis, Sparkle L. Malone, Charles R. Lovell

**Affiliations:** 1 Department of Biology, Wingate University, Wingate, NC 28174, USA; deb.davis@wingate.edu or dadavis26@gmail.com; 2 Department of Biological Sciences, Florida International University, Miami, FL 33199, USA; smalone@fiu.edu; 3 Department of Biological Sciences, University of South Carolina, Columbia, SC 29208, USA

**Keywords:** drought, diazotrophs, salt marsh

## Abstract

Drought has many consequences in the tidally dominated *Spartina* sp. salt marshes of the southeastern US; including major dieback events, changes in sediment chemistry and obvious changes in the landscape. These coastal systems tend to be highly productive, yet many salt marshes are also nitrogen limited and depend on plant associated diazotrophs as their source of ‘new’ nitrogen. A 4-year study was conducted to investigate the structure and composition of the rhizosphere diazotroph assemblages associated with 5 distinct plant zones in one such salt marsh. A period of greatly restricted tidal inundation and precipitation, as well as two periods of drought (June–July 2004, and May 2007) occurred during the study. DGGE of *nif*H PCR amplicons from rhizosphere samples, Principal Components Analysis of the resulting banding patterns, and unconstrained ordination analysis of taxonomic data and environmental parameters were conducted. Diazotroph assemblages were organized into 5 distinct groups (R^2^ = 0.41, *p* value < 0.001) whose presence varied with the environmental conditions of the marsh. Diazotroph assemblage group detection differed during and after the drought event, indicating that persistent diazotrophs maintained populations that provided reduced supplies of new nitrogen for vegetation during the periods of drought.

## 1. Introduction

Salt marshes of the Atlantic and northern Gulf of Mexico coasts of temperate North America are highly productive [1,2,3,4] and commonly dominated by the smooth cordgrass, *Spartina alterniflora*. In these systems, *Spartina* grows in extensive, often monotypic stands [1]. Several other plant species are also present, growing in distinct zones based on edaphic conditions, minor differences in elevation, and interspecific competition [2,3,4]. Semi-diurnal tides maintain moderate levels of interstitial solutes in the sediments of these systems [5,6,7,8], contributing to their high plant biomass and productivity.

Nitrogen frequently limits primary productivity in salt marshes [9,10,11] and plant-associated diazotrophs provide much of the ‘new’ nitrogen in marsh systems that lack riverine freshwater inputs [12,13,14]. Diazotrophic *Bacteria* and *Archaea* are very active in the rhizospheres of salt marsh plants [14,15,16,17] and this activity is tightly coupled to the photosynthetic activity of these macrophytes and to the decomposition of dead plant biomass [14,17,18]. The assemblages of diazotrophs associated with the rhizospheres of salt marsh plants are diverse, mostly novel and mainly belong to the classes α-, β-, γ-, δ-, and ε-*Proteobacteria* [19,20,21,22,23,24,25,26]. These assemblages consist of mixtures of ubiquitous (non-responsive to season or plant host type), seasonally responsive, and plant host specific organisms [27,28]. The assemblages are relatively stable to at least some types of perturbations as long and short-term fertilization and above ground plant biomass removal experiments resulted in only minor changes in the *Spartina* rhizosphere diazotroph assemblage [23,29,30,31]. DNA sequences specific to numerous rhizosphere diazotrophs have been recovered in multiple studies spanning more than a decade and including various marsh conditions [6]. This indicates that these organisms consistently maintained detectable populations in the rhizospheres.

Hydrology is a major driver of species composition in salt marshes and drought can have a significant effect on the structure and function of these coastal ecosystems [32]. Increased average temperatures and reduced precipitation lead to prolonged dry conditions and frequent droughts. These conditions have resulted in mass diebacks in Atlantic coast and Gulf coast marshes [33,34]. Macro and meiofauna could also be negatively impacted by drought and inadequate tidal flooding [32], but the effects of these conditions on microbial communities in salt marsh sediments are not known. The impact of increasingly severe conditions might be magnified if the plant associated diazotrophic bacteria are strongly affected due to the importance of these organisms to marsh productivity.

It will become increasingly important to understand how and why diazotroph assemblages change in response to environmental conditions. Global change is predicted to produce rising mean sea levels, increases in average global temperatures, and reduced frequency of precipitation events in some areas [35,36]. These changes have negatively affected coastal salt marshes worldwide and are especially important to consider due to the large contributions of these ecosystems to coastal productivity. Interannual variability in mean sea level results in fluctuations in tidal heights, which also negatively affect coastal marshes [37,38]. Lowered local sea levels result in insufficient tidal flooding, with concomitant increases in sediment porewater salinities and strong negative impacts on above ground plant productivity. Tidal deficiency displaces marsh plants and changes species diversity when freshwater zones become mesohaline [34].

A 4-year study undertaken to investigate how diazotroph assemblages associated with highly stable plant zones varied, and to determine if changes in environmental conditions in the marsh had any effect on assemblage composition captured a period of unusually low tides and two drought events. This provided a rare opportunity to determine the effects of drought on diazotroph assemblages in situ and on the drivers of coastal ecosystem structure and function. The results of this study indicate that portions of the diazotroph assemblage persisted during and after a drought event and significant changes in the plant zone associated assemblages were correlated with abiotic and soil chemistry parameters. These data provide much needed information on the effects of frequent drought events on salt marsh diazotrophy, the foundation of marsh productivity. Increasing frequency of drought has immediate effects on acute marsh dieback which is expected to limit the ability of intertidal marshes to accommodate expected rising sea levels [39].

## 2. Materials and Methods

### 2.1. Study Area

**Figure 1:**
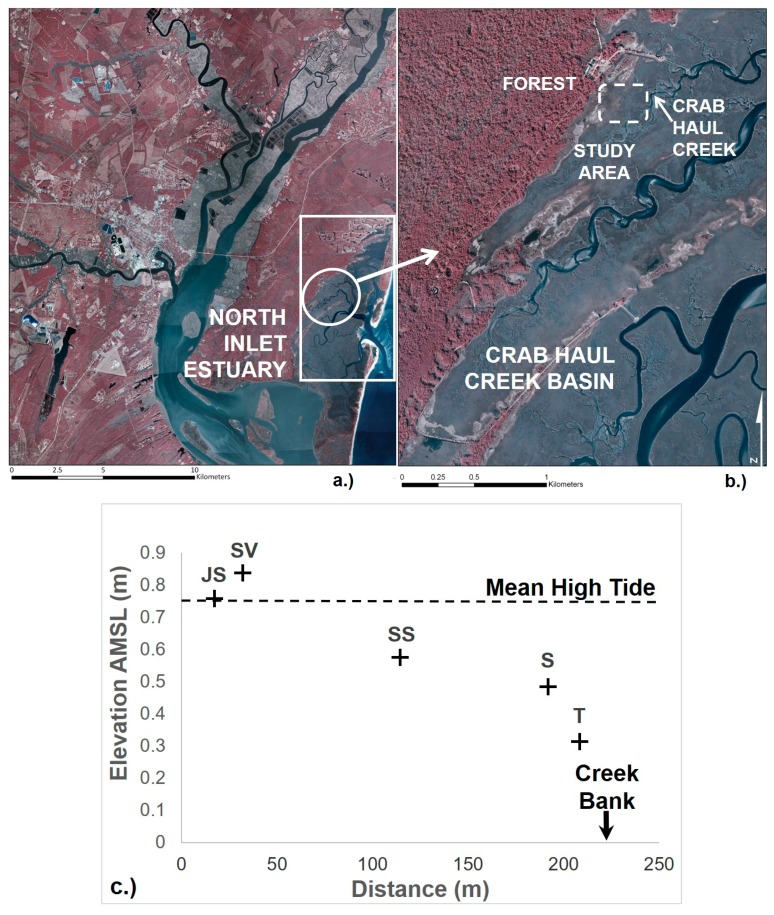
(**a**) Color (false-color) infrared (CIR) aerial photograph of North Inlet Estuary showing Crab Haul Creek Basin (circled area); (**b**) CIR aerial photograph of Crab Haul Creek Basin showing study area, including forest edge and Crab Haul Creek (original CIR image from www.northinlet.sc.edu); (**c**) Vegetation zone elevation differences above mean sea level (AMSL) and distance of each sampling location from the forest edge. Vegetation zone designations: *Juncus roemerianus* stand (JS); *Salicornia virginica* (SV); mixed plant zone of co-occurring *S. virginica* and short form *Spartina alterniflora* (SS); short form *S. alterniflora* (S); and tall form *S. alterniflora* (T). This is an amendment of an original image that was adapted with permission from reference [27]. Copyright 2011 Springer.

The Crab Haul Creek Basin (33°20′ N, 79°12′ W; Figure 1a,b) in North Inlet estuary is a tidally dominated marsh located near Georgetown, SC, USA. The basin is within the humid subtropical climate zone, with a mean annual temperature of 18 °C and receives 1429 mm of precipitation on average annually [40]. With a semi-diurnal tidal range averaging 1.5 m [41], vegetation occurs in distinct mixed and monotypic zones where the distribution of vascular plants is correlated with marsh elevation and governed by biotic and edaphic environmental conditions [5,42]. The study site extended from the terrestrial biome to the edge of Crab Haul Creek (~200 m; Figure 1a,b), across the 5 distinct marsh vegetation zones. At higher elevations of the marsh, monotypic stands of black needlerush (*Juncus roemerianus* Scheele; JS) occurs along the interface of the terrestrial biome with the marsh proper (Figure 1c). The next major vegetation zone consists of monotypic stands of the perennial glasswort (*Salicornia virginica*; SV) that transitions into a mixed zone of co-occurring *S. virginica* and short growth form smooth cordgrass (*Spartina alterniflora* Loisel; SS). Closer to the creekbank are monotypic stands of short growth form *S. alterniflora* (S) and tall growth form *S. alterniflora* (T). Zones were selected and sampled to provide strong contrasts in porewater chemistry and the types of vegetation present.

From 2003–2007, vegetation zones were sampled to evaluate the composition of diazotroph assemblages associated with the plants in these zones. While the climate parameters over the study period were largely within the normal range, drier than normal conditions occurred in 2004 and 2007. The Palmer drought severity index (scPDSI) was used to show the onset, duration, and severity of drought conditions. Monthly scPDSI data made available through the Western Regional Climate Center ranges from −6.0 to 6.0. The number shows the magnitude and the sign denotes (+) wetter than average or (−) drier than average conditions for a location based on historical climate and sensitivity to changes in water availability [43,44,45]. Values of scPDSI between −0.4 and 0.4 denote average conditions, and absolute values greater than 4 represent extreme conditions. Unlike earlier versions of the PDSI, extreme conditions occur based on the history of the location and are not determined relative to a default location [43], which allows for more exact comparisons between location and times, and is a more accurate index compared with PDSI for extreme events [43]. A moderate drought (−3 ≥ scPDSI ≤ −2) occurred in 2004 (June and July) and a severe drought (scPDSI ≤ −3) occurred in 2007 (May–December) along the coast, which provided an opportunity to observe variations in community dynamics in response to drought conditions.

### 2.2. Sample Collection

The five vegetation zones within the study area each contained 6 sampling plots located along a 7 m sampling line that was parallel to Crab Haul Creek and marked with gardening stakes. Plots were positioned in the center of vegetation zones to avoid transitional areas between zones and to facilitate detection of zonal characteristics. Rhizosphere samples were collected from each plot (six samples per zone), in the spring and fall of most years (i.e., September 2003, May and August 2004, May and September 2005, May and July 2006, and May 2007), by coring within each of these plant zones. These dates were chosen to sample the diazotroph assemblages associated with zones at the beginning and end of the growing season when rates of diazotrophy are highest [46]. Samples taken in July 2006 were included because samples from later in the year were unavailable. The sediment cores were collected within the plots using corers for DNA extraction (2.4 cm diameter by 6 cm length corers) and acetylene reduction assay (1.5 cm diameter by 8 cm length corers) as described previously [6,27]. Sediment cores collected for the measurement of acetylene reduction rates were used to prepare slurries in 40 mL serum vials with sterile butyl rubber septa. Ten grams of sediment and 10 mL of sterile artificial seawater (34‰ salinity) were added to the vials, and 1.5 mL acetylene was injected into the 15 mL vial headspace. Assays were performed as previously described by Davis et al. [14,17,27].

Sippers for porewater collection were established at 1 m intervals, at the center of each plot (6 sippers per plant zone). Porewater samples were collected (using methods described in Davis et al., 2011) within 1 h of low tide on each sampling date [47] for the measurement of pH, salinity and concentration of soluble sulfide [48], nitrate [49], nitrite and ammonium ions [50,51]. Sediment temperatures were obtained using a digital thermometer (Fisher Scientific, Pittsburgh, PA, USA). Tide data was obtained from the National Oceanic and Atmospheric Administration (NOAA, Silver Spring, MD, USA) Tides and Currents site [52]. Data from the nearest station (Oyster Landing, North Inlet Estuary, SC, Station 8662245) were used to document tidal patterns.

### 2.3. Sample Processing

#### 2.3.1. DNA Purification

DNA was extracted and purified from each rhizosphere sample using the direct lysis procedure of Lovell and Piceno [27,53]. Prior to PCR amplification, extracted DNA was further purified using the Promega Wizard Clean-up Kit (Madison, WI, USA). For the 2005 and 2006 samples an extra step of preparative agarose gel electrophoresis was required for additional DNA purification. Extracted DNA was electrophoresed through a 0.8% agarose gel in 1× TBE (pH 8.3; 89 mM Tris-HCl, 89 mM Boric acid, and 5 mM Disodium Ethylenediamine Tetraacetate (EDTA)). The DNA was excised from the agarose gel and extracted using the Promega Wizard SV Gel and PCR Clean-up Kit (Madison, WI, USA).

#### 2.3.2. *nif*H Amplification

PCR amplification was carried out using 4 U Taq DNA polymerase in 1× Taq buffer (containing 1.5 mM MgCl_2_) (Qaigen, Valencia, CA, USA) in a 100 µL reaction mixture containing 25 ng of DNA template, 800 µM of each deoxynucleotide triphosphate (New England Biolabs, Ipswich, MA, USA), 0.5 pmol µL^−1^ of each primer (MWG Biolabs, Huntsville, AL, USA) and 40 mg bovine serum albumin (NEB). The *nif*H primers used (Forward primer, *nif*H F; Reverse primer, *nif*H R) [24,54] contain the artificial nucleotides P [55] and K [56]. The use of these artificial nucleotides reduces degeneracy and occurrence of spurious products. PCR amplification was performed using the program described by Davis et al. [27]. Ninety microliters of the reaction mixture were concentrated by isopropanol precipitation. Amplicons were recovered by centrifugation for 30 min at 10,000× *g*, washing in 70% ethanol and recovery in 10 μL of TE (pH 8.0; 10 mM Tris-HCl, 1 mM EDTA).

#### 2.3.3. Denaturing Gradient Gel Electrophoresis

The GC clamp *nif*H amplicons were electrophoresed on denaturing gradient gels (DGGE) as described by LaRocque et al. [57]. Gels were 1 mm thick 6.5% polyacrylamide with a 78–89% denaturant gradient, where 100% denaturant contains 7 M urea and 40% formamide. Gels loaded with 10 µL of *nif*H amplicons in each lane were run for 1900 Vh at 48 °C in a Bio-Rad DCode universal mutation detection system (Bio-Rad Laboratories, Inc., Hercules, CA, USA). Standards used for gel comparisons included GC clamped *nif*H amplicons of *Klebsiella pneumoniae*, *Sinorhizobium meliloti*, and *Azospirillum lipoferum*. The gels contained an artifact band in each lane that was likely single stranded DNA [54] and was used as an additional reference. Gels were stained with 15 µL of SYBR Gold (Molecular Probes, Eugene, OR, USA) in 200 mL 1× TE for 30 min and images were acquired using an Alpha Imager 2000 (Alpha Innotech Corp., San Leandro, CA, USA) at an aperture setting of 1.2 aperture and zoom setting of 19.

### 2.4. Statistical Analysis

#### 2.4.1. Diazotroph Community Composition: DGGE Band Pattern Analysis

Gel analysis followed the methods of Davis et al. [27,28] and were accomplished using GelCompar II software (BioSystematica, Devonshire, UK). After analysis of the total bands for all samples, the bands were assigned an ultimate band number (UBN) [28] for a total of 45 identified band positions. Presence or absence of each of the 45 band positions for every lane (each replicate) was determined and the binary data (from 196 individual lane samples) were compiled for statistical analysis (Supplementary materials, Table S1). The Multi Variate Statistical Package 3.13 g (MVSP) (Kovach Computing Service, Wales, UK) [58] was used to statistically analyze the presence/absence binary data. Principal Components Analysis (PCA) of the presence/absence data using Kaiser’s Rule was performed and results were plotted both with and without Euclidean bi-plot. The Euclidean bi-plot provided eigenvectors indicating DGGE bands that controlled clustering. Data by date and data by plant zone were compared and figures were produced for all comparisons. MVSP produces a sign condition (+/−) causing some groups to cluster to the left of the main axis whereas a similar data group clusters to the right of the main axis. It has been previously determined that this is an artifact of the program and not a result of the data [28], therefore the data were corrected for the axis shift. UPGMA analysis was not carried out as the results from this analysis tend to correlate with PCA analysis (based on overall clustering) and UPGMA does not provide information on the impact of individual bands on sample clustering [28]. A distance measurement between clusters was used to establish the significance of clustering [27,28]. A two-tailed student’s *t*-test (α = 0.001) was used to determine the significance of the distances between the various date and plant type groups. Data used in this analysis has been archived on the Knowledge Network for Biocomplexity [59].

Bands present in the banding patterns of DGGE gels having very obvious changes in the banding patterns (May and September 2005, and May and July 2006) were sampled for sequencing using previously described methods [24,28,60]. Twenty-one sequences were deposited in the National Center for Biotechnology Information (GenBank accession numbers HM750261−HM750281). Nucleotide sequences were imported into ClustalX version 2.0.12 (The Pennsylvania State University, University Park, PA, USA) [61,62], and primer sequences were removed after alignment. The Molecular Evolutionary Genetics Analysis software version 7.0 (MEGA7) [63] was used, after manual conversion of the sequencesto a format recognizable by that software, to construct a Neighbor-Joining phylogenetic tree with 20 formally described *nif*H reference sequences using the Jukes-Cantor correction for nucleotide sequences, complete deletion of missing data and gaps, and 1000 bootstrap replications. The outgroup taxon used was the *Methanothermobacter thermoautotrophicum nif*H sequence (GenBank accession number X87971). A BLAST study was used to determine suitable sequences for comparison.

#### 2.4.2. Environmental Conditions and Sediment Chemistry

A mixed modeling approach was used to test for significant differences in conditions over the study period to understand how environmental conditions change overtime. This approach was utilized to incorporate variance–covariance matrices explicitly formulated to appropriately account for the random effect of the sample design and the repeated measurement of each experimental unit over time. Temporal models for sediment temperature (°C), tide (m), salinity levels (ppt), and pH included fixed effects for sampling date. Random effects included the interaction between plot and the vegetation zone. Mixed modeling methods were also used to test for differences between vegetation zone for sediment temperature (°C), tide (m), salinity (ppt), pH, ammonia (NH_3_), nitrate (NO_3_^−^), nitrite (NO_2_^−^), and sulfide (S^2−^). Assumptions of normality and homoscedasticity were evaluated visually by plotting residuals and all the statistics were carried out using the program R 3.3.2 [64] and the package *lme4* was used in mixed-effects modeling [65].

#### 2.4.3. Diazotroph Assemblages: Unconstrained Ordination Analysis

Ordination methods were used to delineate diazotroph assemblages and to understand how environmental conditions influence ordination with the R package *vegan* [66,67,68]. Unconstrained ordination transformed species presence/absence data for the species detected by decomposing the total variance into linear components in two main dimensions (i.e., PCA 1 and PCA 2), while preserving maximum information. Diazotroph assemblage groups were defined using a Jaccard dissimilarity index [69] and a hierarchical cluster analysis on the dissimilarities. The R function *hclust* was used to perform the hierarchical cluster analysis [67]. This approach uses the set of dissimilarities. Initially, each measurement period is assigned to its own cluster and then the algorithm proceeds iteratively, at each stage joining the two most similar clusters. At each stage distances between clusters were recomputed by the Lance–Williams dissimilarity update formula according to the particular clustering method being used. Here the *complete linkage* method which finds similar clusters [70] was used. This analysis was necessary to understand partitions in species assemblage groups by defining clusters based on dis-similarities [71]. The aim was to minimize within group variation and maximize between-group variation in order to reveal well-defined assemblage clusters, and reduce the dimensionality of assemblages into a few groups [71]. Data used in this analysis has been archived on the Knowledge Network for Biocomplexity [59].

#### 2.4.4. Drivers of Ordination and Diazotroph Assemblage Presence

Assemblage groups were used in combination with vegetation zones, environmental conditions, and sediment chemistry to understand the variation in the two PCA axes that describe change in diazotroph assemblages over time for each vegetation zone using the function *envfit* [66]. Variables included sediment temperature (°C), tide (m), salinity (ppt), pH, ammonia (NH_3_), nitrite (NO_2_^−^), nitrate (NO_3_^−^), sulfide (S^2−^), vegetation zone, and assemblage group. The significance of variables was assessed using 10,000 permutations of variables to calculate goodness of fit (R^2^) and *p*-values. Regardless of *p*-values, all variables were kept in the model. A *p*-value of 0.15 was used as the cut-off for significance [72]. All analyses were carried out using the program R 3.3.2 [64].

## 3. Results

### 3.1. Anecdotal Evidence for 2004 Drought Event

**Figure 2:**
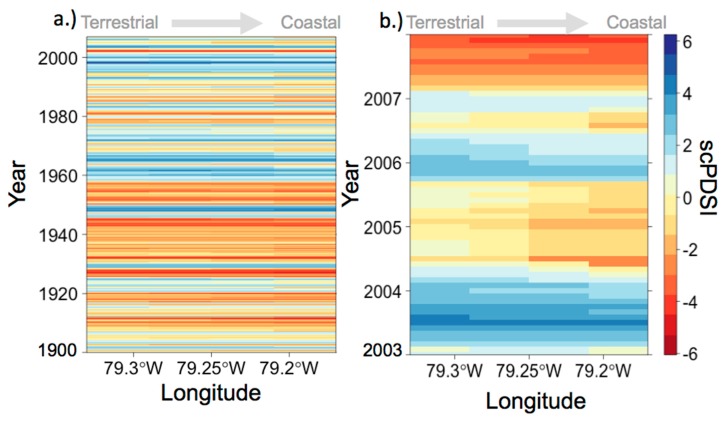
(**a**) The monthly Palmer drought severity index (scPDSI) was used to show the frequency of wetter than average and drier than average conditions for North Inlet estuary and the surrounding coastline. (**b**) Monthly scPDSI over the study period shows that both wetter than average and drier than average conditions did occur with two drought events along the coast, moderate drought conditions in July 2004, and severe drought conditions from May to December of 2007.

During April and May of 2004, the marsh did not experience any tidal inundation due to unusually low tides (personal observation, see archived data for tide measurements [59]). The lack of tidal inundation in the high and mid marsh, higher than average temperatures and moderate drought conditions (Figure 2) resulted in a very dry marsh—drained, dry and cracked sediment, with dead flora and fauna. Dry conditions lasted throughout the entire growing season of 2004, making it difficult at times to collect porewater, especially in the high marsh region closest to the forest edge. In addition, during the years 2004 and 2005 the mean high water relative to mean sea level was at the lowest level it had been in approximately 10 years [37].

### 3.2. Diazotroph Community Structure and Composition

**Figure 3:**
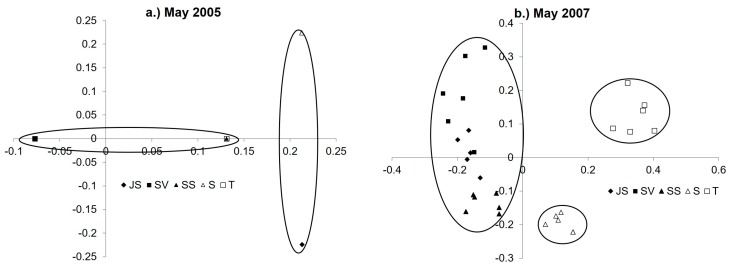
PCA results for dates (**a**) May 2005 and (**b**) 2007 (JS, SV, SS, S and T). Circles denote significance (*p* < 0.001) for clustering. For May 2005 Axis 1 represents 61.3% of the variance and Axis 2 represents 84.8% of the variance. For May 2007 Axis 1 represents 31.2% of the variance and Axis 2 represents 52.1% of the variance. Vegetation zone designations: *Juncus roemerianus* stand (JS); *Salicornia virginica* (SV); mixed plant zone of co-occurring *S. virginica* and short form *Spartina alterniflora* (SS); short form *S. alterniflora* (S); and tall form *S. alterniflora* (T).

**Figure 4:**
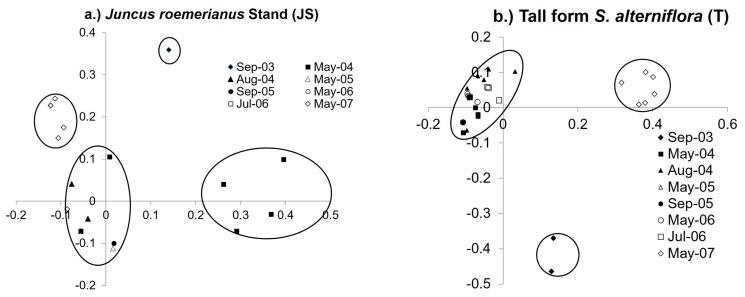
PCA results of (**a**) high marsh *Juncus roemerianus* (JS) and (**b**) low marsh tall form *Spartina alterniflora* (T) for all dates. Circles denote significance (*p* < 0.001) for clustering. For JS Axis 1 represents 27.5% of the variance and Axis 2 represents 50.8% of the variance. For T Axis 1 represents 38.3% of the variance and Axis 2 represents 54.1% of the variance.

PCA analysis was conducted to explore the correlation of the DGGE banding patterns to vegetation zone and sampling dates (Figure 3 and Figure 4 and Supplementary materials, Figures S1 and S2). For September 2003 and May 2007 there was significant clustering (*p* < 0.001) of plant zones based on their location in the marsh (high elevation vs. low elevation, see Figure 1c). In May 2004 high marsh *Juncus roemerianus* stand (JS) and mid-marsh mixed zone of co-occurring *Salicornia virginica* and short form *Spartina alterniflora* Loisel (SS) plant zone samples clustered significantly, high elevation *Salicornia virginica* (SV) plant zone samples clustered separately from all others and low marsh short form *Spartina alterniflora* (S) clustered significantly with tall form *Spartina alterniflora* (T) plant zone samples. The August 2004 SS plant zone samples clustered significantly but samples from all other plant zones grouped together in mixed clusters. May and September 2005, and May and July 2006 samples also clustered significantly together in mixed clusters. The DGGE gels for these dates (2005 and 2006) showed significant changes in the banding patterns when compared to the banding patterns for samples from 2003 and 2007. The number of bands called during gel analysis was much lower for these dates (9−12) than 2003 (35), 2004 (25) and 2007 (27).

The trends in clustering were also evident in the PCA analysis by plant zone. For the JS plant zone all dates (other than September 2003, May 2004, and May 2007) clustered together in a mixed cluster (Figure 4a). The T zone exhibited similar clustering patterns to the JS zone, with September 2003 and May 2007 forming individual clusters and all other dates clustering together in a mixed group (Figure 4b). However, the SV plant zone, September 2003 and May 2007 samples clustered significantly together, and all other dates formed a mixed cluster. The mixed plant zone (SS) samples had a different pattern of clustering; the August 2004 and May 2007 samples formed a significant cluster, and the September 2003 samples clustered together while all other dates formed a mixed cluster. The low marsh S plant zone May 2004 and 2007 samples formed separate significant clusters and all other dates clustered together in a mixed group.

**Figure 5:**
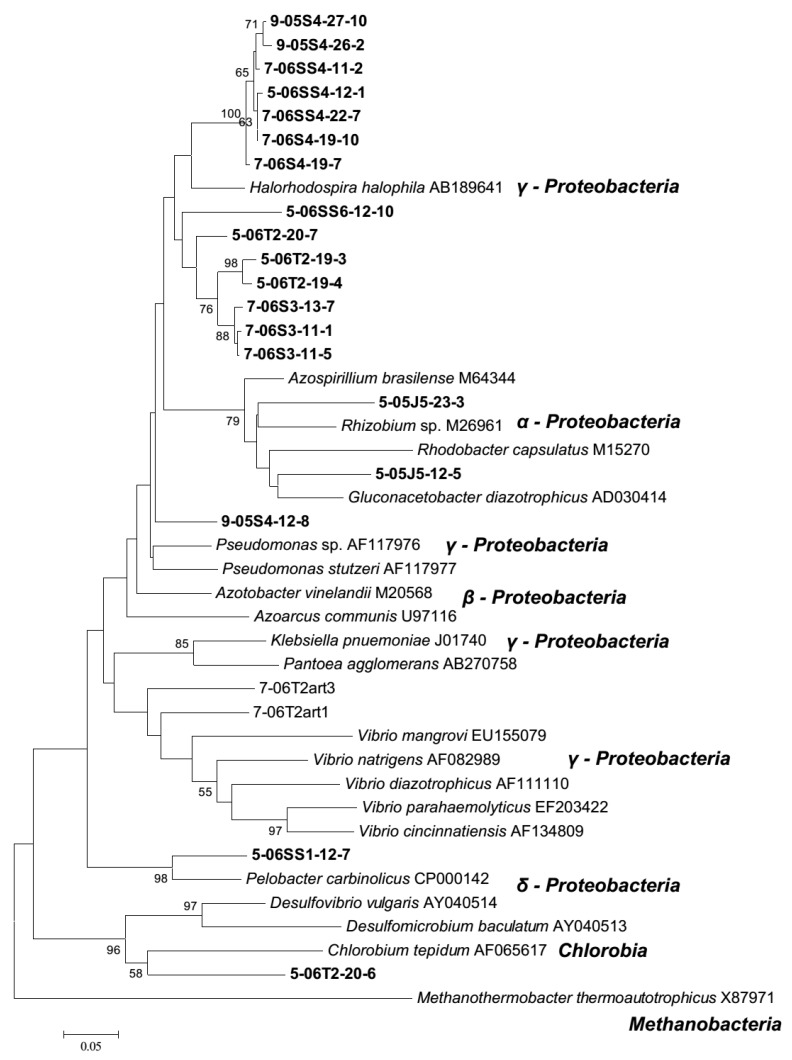
Phylogram of sequences from DGGE band stabs from 2005 and 2006 post drought rhizosphere samples (Nucleotide sequences, Neighbor-Joining, Jukes-Cantor correction, 1000 bootstrap replicates, complete deletion of gaps and missing data). Sequence names depict sampling date (prefix, e.g., 5-05 for month and year), plant zone (e.g., JS for *Juncus roemerianus* stand). Numbers following the prefix in the sequence name are ultimate band number and clone designation. Sequences from corresponding UBNs listed in diazotroph assemblage group 1 (Table S2) are bolded.

DGGE gels with severely reduced numbers of bands in the banding patterns (2005 and 2006) were sampled for nucleotide sequence analysis to identify the diazotrophs that maintained detectable populations in the plant rhizospheres. Not all bands could be sequenced due to low yield and quality of DNA from some samples. Twenty-one sequences were obtained, with at least one sample from each date sequenced and all zones were represented except the *S. virginica* (SV) plant zone. One group (7 sequences total) had less than 6% within-group differences in their nucleotide sequences and are considered the same genomic species (cut off for nucleotide sequences of protein coding marker genes, identical, sensu Venter) [60,73] (Figure 5). This group of sequences were strongly aligned with the *nifH* sequence of *Halorhodospira halophila* (γ-Proteobacteria). Four other sequences grouped with formally described reference sequences, *Rhizobium* sp., and *Gluconacetobacter diazotrophicus* (α-*Proteobacteria*), *Pelobacter carbinolicus* (δ-*Proteobacteria*), and *Chlorobium tepidum* (*Chlorobia*), while the other 10 sequences did not group with any formally described taxa.

### 3.3. Environmental Conditions and Sediment Chemistry

**Figure 6:**
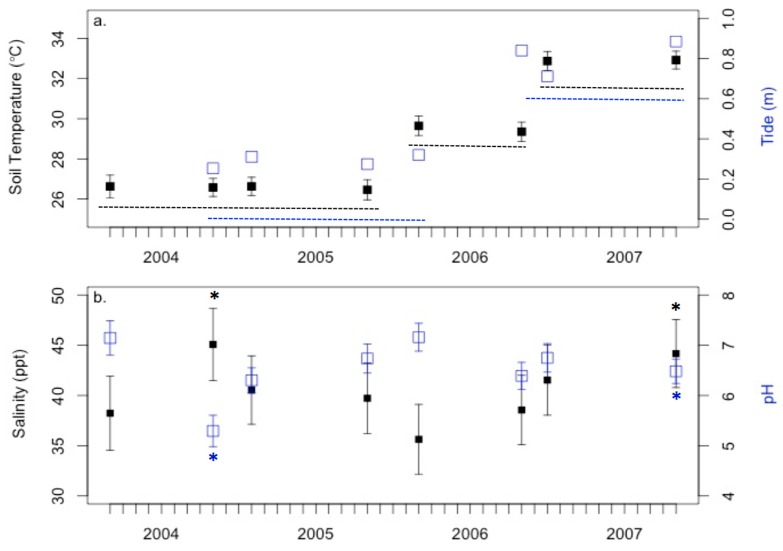
Environmental conditions least square means over the study period. (**a**) sediment temperature (black) and tide (blue) increased over the study periods. Dotted lines indicate which timeframes were similar statistically; (**b**) Salinity (black) and pH (blue) fluctuated and asterisks (*) denote timeframes with statistically high or low conditions. Error bars denote standard errors.

**Figure 7:**
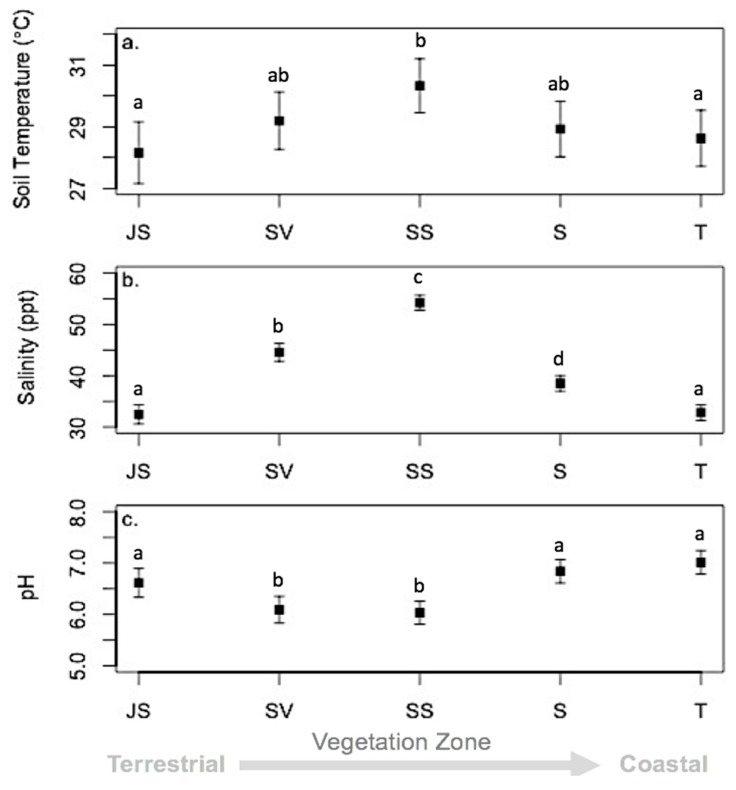
Environmental conditions least square means by vegetation zone. (**a**) Soil Temperature (°C); (**b**) salinity (ppt); and (**c**) pH. (JS = high marsh *Juncus roemerianus*; S = low marsh short form *S. alterniflora*; SV = high marsh *Salicornia virginica*; SS = mid-marsh mixed zone of co-occurring *S. virginica* and short form *Spartina alterniflora*; T = low marsh tall form *S. alterniflora*). Letters denote significant differences between vegetation zones; zones with the same letters are similar to each other but significantly different from zones designated by different letters. Error bars denote standard errors.

Environmental conditions (soil chemistry and abiotic parameters) varied over the study period (Figure 6). Both sediment temperature and tide increased significantly over time (*p* < 0.05; Figure 6a) while salinity and pH fluctuated (Figure 6b). Salinity levels in May 2004 and 2007 were significantly higher (*p* < 0.001) than all other time frames and were statistically similar to each other. Like patterns in salinity, pH levels in May 2004 and 2007 were significantly lower (*p* < 0.001) than all other time frames. Environmental conditions also varied across vegetation zones. Sediment temperatures were highest for the S zone (30 ± 0.44 °C), which was significantly different from the high marsh JS (*p* = 0.01) and the low marsh T (*p* = 0.05) zones (Figure 7a). Salinity was greatest for the S zone, and lowest for the high marsh JS and T zones (Figure 7b). All zones except high marsh JS and low marsh T had significantly different salinity levels. Vegetation zones were clustered into three distinct groups for pH (Figure 7c). The low marsh T zone had the highest pH followed by the high marsh JS and the low marsh S zone. The lowest pH was observed at the SV and the mid-marsh SS plant zones.

**Figure 8:**
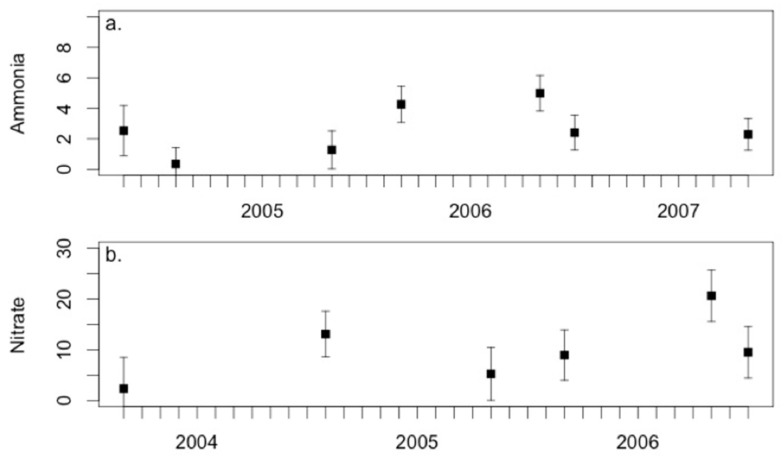

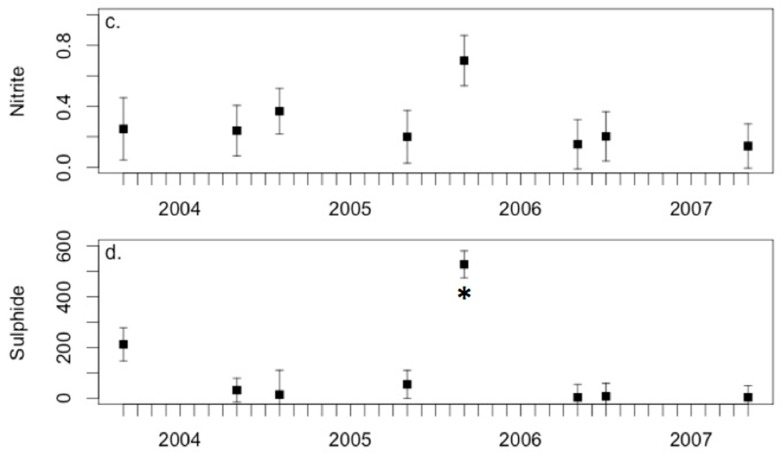
Sediment chemistry least square means over the study period. (**a**) Ammonia; (**b**) nitrate; (**c**) nitrite, and (**d**) sulfide fluctuated over the study period. Asterisks (*) denote timeframes with statistically high conditions and error bars denote standard errors.

**Figure 9:**
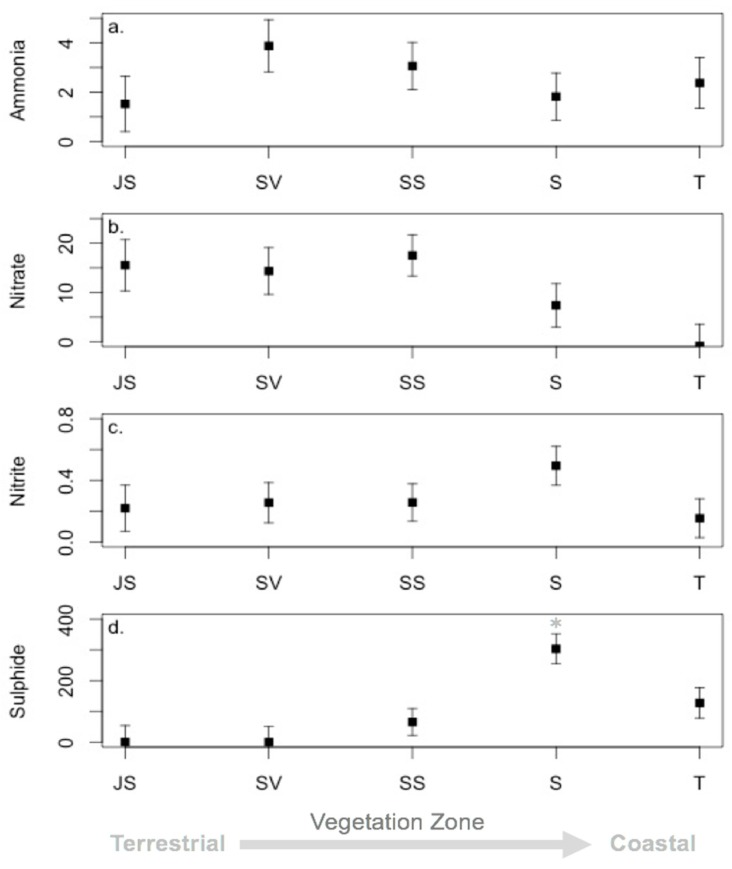
Sediment chemistry least square means by vegetation zone. (**a**) Ammonia; (**b**) nitrate; (**c**) nitrite, and (**d**) sulphide. (JS = high marsh *Juncus roemerianus*; S = low marsh short form *S. alterniflora*; SV = high marsh *Salicornia virginica*; SS = mid-marsh mixed zone of co-occurring *S. virginica* and short form *Spartina alterniflora*; T = low marsh tall form *S. alterniflora*). Asterisks (*) denote significant differences between vegetation zones and error bars denote standard errors.

Although sediment chemistry fluctuated over the study period, there was only one sampling date with significantly different sulfide levels. Sulfide levels in September 2005 were significantly higher than all other time frames (*p* < 0.001; Figure 8). Comparisons of sediment chemistry between vegetation zones also indicated that most zones had similar chemical makeup with significant differences in sulfide between the low marsh S and all other zones (*p* < 0.001; Figure 9). Ammonia, nitrite, and nitrate showed no significant differences over the study period and between vegetation zones. Acetylene reduction was undetectable in the JS plant zone during the 2003 and 2004 samplings (Table S3). Rates of acetylene reduction were higher at the beginning of the growing season (May 2005, 2006 and 2007) in the JS and T plant zones. The same trend was observed in the SV plant zone, except in May 2004 when no acetylene reduction was detected there. The opposite was observed in the low marsh S plant zone, where rates were higher at the end of the growing season, except in May 2006 when higher rates of acetylene reduction were measured than in July 2006. During all sampling dates the highest rates of acetylene reduction were observed in either the SS or SV plant zones; except May 2007, when highest rates were observed in S plant zone. The lowest rates were consistently in the high marsh JS plant zone and low marsh T plant zone.

### 3.4. Diazotroph Assemblages

**Figure 10:**
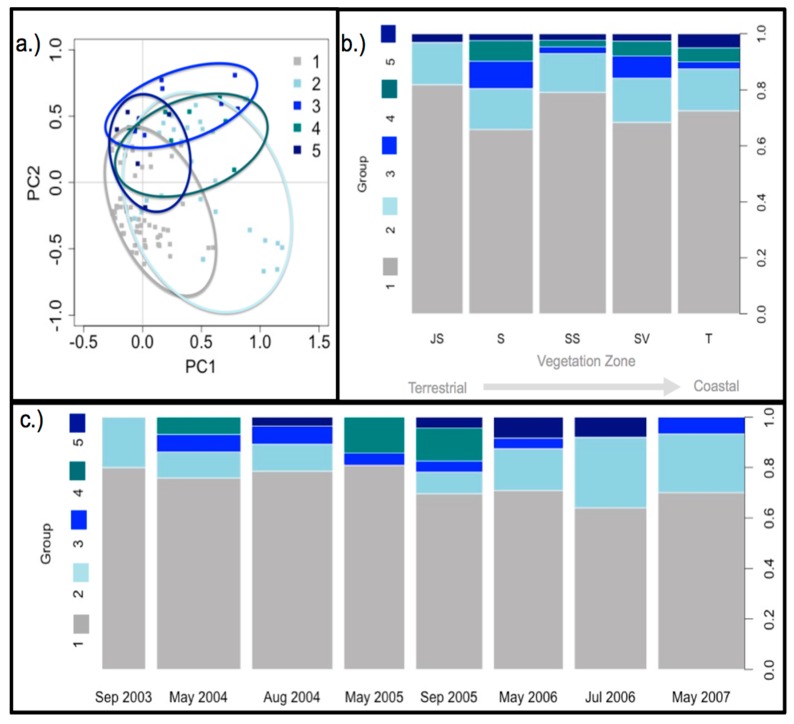
(**a**) Ordination and (**b**) variation in assemblage groups by vegetation zone (JS = high marsh *Juncus roemerianus*; S = low marsh short form *S. alterniflora*; SV = high marsh *Salicornia virginica*; SS = mid-marsh mixed zone of co-occurring *S. virginica* and short form *Spartina alterniflora*; T = low marsh tall form *S. alterniflora*) and (**c**) over time. The width of each block is proportional to the number of core samples with successful DNA isolation and subsequent DGGE gel analysis.

Community assemblage data was clustered into 5 distinct assemblage groups that varied over time and across vegetation zones (Figure 10; Supplementary materials Table S2). The UBNs in each diazotroph assemblage group (Table S2) were a collection of taxonomic groups defined during DGGE analysis. The 5 groups contained similar UBNs as is evident in the ordination plot of the groups (Figure 10a), however distinct dissimilarities were significant enough for delineation. Assemblage group 1 contained the most common UBNs (UBNs of that group were also present in all others) and was the only group present consistently throughout the study period. Prior to 2005, all assemblage groups were present. Between 2005 and May 2006, assemblage group 1 was present in most plant zones (Figure S3) with groups 2 and 4 reappearing in and after September 2005. Assemblage groups 1, 2, and 5 were present in all vegetation zones (Figure 10). Assemblage group 4 was present in all zones except the high marsh JS. Assemblage group 3 was present in higher proportions in the low marsh S and the mid-marsh SS. Similar to patterns observed in vegetation zones, assemblage group 1 was the dominant group detected over the study period (Figure 10). Prior to the first drought in May 2004, groups 1, 2, 3 and 4 were detected. After the first drought, August 2004, assemblage group 5 appears in the assemblage, is undetected in May 2005, reappears in September 2005 and is detected through July 2006. By May 2007 groups 1, 2, and 4 were the dominant groups detected across sites.

### 3.5. Drivers of Ordination and Diazotroph Assemblage Presence

Assemblage groups and vegetation zones were the strongest drivers of ordination (Table 1). Assemblage groups accounted for the largest amount of variation explained (R^2^ = 0.41), followed by vegetation zone (R^2^ = 0.07), sediment chemistry (R^2^ = 0.01−0.02), and environmental conditions (R^2^ = 0.067). Although assemblage group and vegetation zone were the only significant parameters at an 85% significance level, interactive effects between environmental conditions and subsequent changes in sediment chemistry are likely influencing shifts in the assemblage group presence detected in the rhizosphere over the study period (Supplementary materials, Figure S3). The prominence of assemblage group 1 occurs in May 2004, with dry conditions. Following a wetter than average period (2005–2006; see also Figure 2), assemblage groups 5 appears in vegetation zones. Environmental conditions were not adequately captured in the study design to develop informative models for diazotroph assemblages.

## 4. Discussion

From September 2003 to May 2007 the salt marsh system in Crab Haul Creek Basin, North Inlet, SC went through several dramatic changes. The moderate drought event in combination with the lack of tidal inundation during the growing season of 2004 had a significant effect on the marsh ecosystem that was easily observable. In addition, during the years 2004 and 2005 the mean high water relative to mean sea level was at the lowest level it had been in approximately 10 years [37]. Droughts lead to substantial changes in saltmarshes, and in the period of this study, this marsh landscape experienced two such events (June–July 2004, and May 2007). The lowered sea level had a great effect on flooding frequency of the marshes, consequently resulting in higher porewater salinity especially during warmer summer months. Increased salinities in turn results in lower plant productivity [38]. Lack of tidal inundation and drought conditions in the system had lasting effects that were obvious in the porewater chemistry, and the shifts in the composition of the diazotroph assemblages.

### 4.1. Sediment Chemistry is Somewhat Affected by Environmental Conditions

Porewater chemistry exhibited clear differences among ‘drought’ years and ‘normal’ years. Drought conditions led to significantly higher sediment temperatures, and salinity, and lower pH (Figure 6), however over the four years of sampling there were also significant similarities in porewater chemistry parameters based on location in the marsh, season, and plant zone. Higher salinity levels in the mid-marsh zones were most likely due to being exposed for longer periods in between tides and evaporation. The high marsh and low marsh salinities were significantly different from that experienced in the mid-marsh, as the high marsh is inundated less frequently, and the low marsh is constantly in contact with the creek. Similar patterns were observed with sediment temperatures; however, pH was significantly lower in the mid-marsh than the high and low marsh environments. There were no significant patterns observed with porewater chemistry except for significant levels of sulfide in the short form *Spartina alterniflora* (S) zone and during the 2006 growing season.

Near neutral pH was typical for this system, except during the drought year (2004) and the year immediately following (2005). Drought conditions and a lack of tidal inundation resulted in drained sediments which were more aerated and likely resulted in formation of sulfuric acid from abiotic and microbial oxidation of pyrite and sulfide [74]. In 2005 pH values were close to neutral but lower than those recorded in all other years in the data set. Concentrations of soluble sulfide were lowest during the drought year, consistent with the increased oxidation of sulfide, however in September 2005 they were exceptionally high. This indicates that either rates of sulfate reduction were also exceptionally high during that growing season or a prolonged accumulation of sulfide occurred in the sediment. High concentrations of sulfide inhibit uptake of ammonium by plants [75], possibly contributing to the high concentrations of soluble ammonium recorded in all plant zones on that sampling date. These concentrations could also be indicative of high rates of microbial decomposition of dead plant material. Considerable decomposition of dead *Spartina* biomass occurs relatively quickly, resulting in losses of up to 61% of dry weight in 23 days [76]. However, if conditions in the sediment were not conducive to microbial activity, the high concentrations of soluble ions could suggest a delayed response to an increase in dead plant material left over from a stress event. Acute stress events, such as drought, have resulted in mass dieback in southeastern US marshes [33,77] and this drought event resulted in large amounts of dieback observed in all zones of this marsh system (Davis, personal observation). High concentrations of ammonium ion and increased aeration of drained sediments (during the drought years) may have increased rates of nitrification resulting in higher concentrations of nitrate and nitrite [78].

Throughout this period nitrogen fixation was detected (through ARA activity) and the highest ARA rates recorded in this data set occurred in the growing season of 2005 which was after the drought event. During the drought acetylene reduction activity (a proxy for nitrogen fixation) was not detectable in the high marsh and was lower than normal in the mid-marsh mixed plant zone (co-occurring short form *Spartina alterniflora* and *Salicornia virginica,* SS), and low marsh S plant zone. However, in the low marsh tall form *Spartina alterniflora* (T) plant zone, the only marsh zone that remained wet during the drought period, rates of acetylene reduction were typical for that zone when compared to other dates. Acetylene reduction rates increased to typical levels as conditions in the marsh improved with higher rates observed at the beginning of the growing season. The high rates of acetylene reduction observed in the SV and SS zones are typical for this system [6].

### 4.2. Diazotroph Assemblages

The patterns of clustering in PCA analysis observed for the September 2003 and May 2007 samples were typical for this system. Previous studies observed clustering of samples from plant zones that contained the same dominant macrophyte or were located at a similar elevation in the marsh [6,27,28]. The data obtained in this study indicate that the diazotroph assemblages associated with the rhizospheres of these salt marsh plants are similar to those previously documented. The diazotroph assemblages associated with salt marsh plant rhizospheres are somewhat stable. Only minor changes in the assemblages were observed when long and short-term fertilization and above ground biomass removal experiments were performed [29,30,31,79]. The drastic difference in the diazotroph assemblages in 2005 and 2006 as observed in DGGE band determination and deemed significant by PCA cluster analysis, indicate that the moderate drought event that occurred in 2004 directly affected these assemblages. The mass mortalities of flora and fauna that were evident across the marsh landscape were immediate, yet the assemblages remained intact and changes were not immediately evident.

Identification of sequences sampled from DGGE gels with DNA from May and September 2005, and May and July 2006, provided some insight into the types of diazotrophs that were present in the assemblages after the drought event. Two of the sequences grouped strongly with presumptive anaerobes, the fermentative δ-proteobacterium, *P. carbinolicus*, and the anaerobic photolithoautotroph, *C. tepidum*. Seven identical (sensu Venter [60,73]) sequences also formed a group with the anaerobic photoautotroph γ-proteobacterium, *H. halophila*. The other 12 sequences grouped strongly with presumptive oxygen utilizers, indicating that the assemblages shifted from having an equal representation of presumptive anaerobes and oxygen utilizers [6]. One of these presumed oxygen utilizing taxa grouped strongly with *G. diazotrophicus*, an α-proteobacterium that fixes nitrogen at low pO_2_ and low pH [80]. These taxonomic groupings are representative of those previously observed in this salt-marsh system [6].

### 4.3. Persistent Diazotrophs Maintain Marsh Dynamics Post Drought Event 

Davis et al. [27] and Gamble et al. [28] have determined that the diazotroph assemblages associated with the rhizospheres of salt marsh plants respond to ordinary seasonal changes, however, such a radical shift in the composition of the assemblage has not been seen before. The long-term effects of water deficiency would change the oxic/anoxic transition in soils and could cause dramatic changes in the composition of the microbial community [81]. Increasing the depth of the oxic layer of the sediment would reduce the strict anaerobe assemblage and decrease rates of anoxic and suboxic processes unique to microorganisms, such as nitrogen fixation. The recovery of sequences affiliated with photoautotrophic anaerobes and oxygen-utilizing diazotrophs that fix nitrogen under low concentrations of oxygen and acidic pH suggests that the diazotrophs present in the post-drought assemblages have maintained detectable populations based on their abilities to resist the drastic changes in edaphic conditions that occurred due to the lack of water. The presence of seasonally responsive diazotrophs suggests that these organisms can maintain low (undetectable by the methods used here) populations under severe environmental conditions, responding rapidly when conditions become conducive for growth. The presumably reduced levels of substrates in the sediment (due to lower plant productivity) could explain the recovery of *nifH* sequences similar to those of photoautotrophic species belonging to the presumptively anaerobic portion of the assemblage.

Diazotroph assemblages were sorted into 5 distinct groups based on their dissimilarities, and ordination analysis indicates how the assemblage changed over seasons and differed between plant zones (Figure 10b,c and Figure S3). Assemblage group 1 was the most stable group as it was present in all zones, at all dates of sampling, and also accounted for the greatest proportion of the assemblages when grouped by plant zone or sampling date. Assemblage group 2 was also quite prevalent throughout the study period; it was present in all plant zones and most sampling dates. When the diazotroph assemblage groups are teased out per zone and assemblage, a clear pattern appears (Figure S3) where group 1 and 2 dominate during ‘normal’ environmental conditions and during drought conditions, however during post-drought conditions an increase in groups 3, 4, and 5 can be observed throughout the various assemblages. This indicates that some diazotrophs persist and maintain detectable populations during and after the drought event, however diazotrophs that are not usually active and/or present in high numbers begin to significantly contribute within the diazotroph population. All the sequences obtained for phylogenetic analysis (Figure 5) were from bands listed (in bold in group 1 only, Table S2) in diazotroph assemblage groups. DNA from other bands was not successfully isolated, providing only a mere glimpse into the possible taxonomic groups to which these microorganisms belong.

Low but detectable rates of acetylene reduction also indicate that diazotrophs present in the assemblages were actively fixing nitrogen under the distressed conditions. In the post-drought sampling years, acetylene reduction rates were the highest recorded, indicating that the recovering assemblage could fix nitrogen very effectively. The physiological mechanisms by which salt marsh diazotrophs withstand acidic pHs, high salinities and low moisture conditions to maintain detectable populations and activity are unknown. Microorganisms acclimate to stressful conditions by reallocating their resources from growth to survival pathways, many ultimately entering a dormant status if the stressful conditions do not otherwise kill them [82]. Diazotrophs capable of tolerating stressful conditions in salt marsh sediment and fixing nitrogen would be essential in maintaining nitrogen availability for plant uptake, thereby contributing significantly to plant productivity. Diazotrophs and rates of nitrogen fixation are tightly coupled to photosynthetic activity of the marsh grasses [14,46]. It is not clear whether the plants require the microorganisms to be productive, but this additional source of ‘new’ nitrogen in a nitrogen limited system would certainly seem conducive to ecosystem function. 

### 4.4. Implications and Future Studies

Due to the oscillation of mean high water relative to mean sea level, tidally-dominated salt marsh ecosystems will be subject to frequent perturbations of flooding or infrequent tidal inundation leading to drought conditions [37,38]. These episodic perturbations have very obvious effects on flora and fauna in the marshes but the direct effects on microbial communities associated with these ecosystems would not have been predicted to be very significant [81]. The data from this study shows that this expectation is not quite correct. The effects on microbial communities may not be immediate but are profound and lasting. The dramatic reduction in the detectable rhizosphere diazotrophs lasted for 2 years even though the marsh landscape seemed to have entered a recovery phase in the very next growing season as conditions improved. The recovery of previously defined ecologically significant sequences in the post-drought assemblages indicates their ability to maintain populations under severe conditions. It is reasonable to assume that these diazotrophs in this nitrogen-limited yet highly productive ecosystem are of fundamental importance to the vitality of this ecosystem. Further studies will be required to determine the physiologies of these organisms, their capacities for nitrogen fixation and their physiological responses to stressful conditions. Manipulated greenhouse experiments will also be necessary to gather informative data that can be used to determine a model of the effect these conditions have on diazotroph assemblages. Such information would provide much needed perspective on the role of microorganisms during severe weather-related events.

## Figures and Tables

**Table 1:** Drivers of ordination.

Parameters		PC1	PC2	R^2^	*p*-Value
Tide (m)		−0.8349	0.5504	0.01	0.894
Sediment Temperature (°C)		−0.4182	0.9084	0.01	0.931
Salinity (ppt)		−0.3844	−0.9232	0.01	0.782
pH		0.5676	0.8233	0.02	0.461
Ammonia		−0.4668	−0.8844	0.01	0.698
Nitrate		−1.0000	−0.0023	0.02	0.449
Nitrite		0.8412	−0.5408	0.02	0.326
Sulfide		0.9900	0.1414	0.02	0.352
Group	1	−0.1079	−0.1349	0.41	0.001
	2	0.4435	0.1412		
	3	0.0883	0.6255		
	4	0.4232	0.4104		
	5	−0.0277	0.3129		
Vegetation Zone	JS	0.0159	−0.1705	0.07	0.131
	SAL	−0.0235	−0.0705		
	SS	−0.0573	−0.0658		
	S	0.0731	0.1548		
	T	0.0402	0.0563		

## Supplementary Materials

The following are available online at www.mdpi.com/link, Figure S1: PCA results for dates (a) September 2003; (b) May 2004; (c) August 2004; (d) September 2005; (e) May 2006; and (f) July 2006, Figure S2: PCA results of (a) *Salicornia virginica*; (b) mid-marsh short form *S. alterniflora* and *S. virginica*; and (c) low-marsh short form *S. alterniflora*, Figure S3: Variation in assemblage groups by zone, Table S1: Number of samples used per zone for each date in DGGE analysis, Table S2: Ultimate Band Number (UBN) per diazotroph assemblage group identified through ordination, Table S3: Acetylene reduction assay results.

Supplementary File 1
