# Supplementary material for: Responses of Salt Marsh Plant Rhizosphere Diazotroph Assemblages to Drought
Source: Microorganisms. 2018 Mar 15;6(1):27. doi: 10.3390/microorganisms6010027 (PMC5874641; doi:10.3390/microorganisms6010027)
Supplement: Supplementary File 1 [file microorganisms-06-00027-s001.pdf]

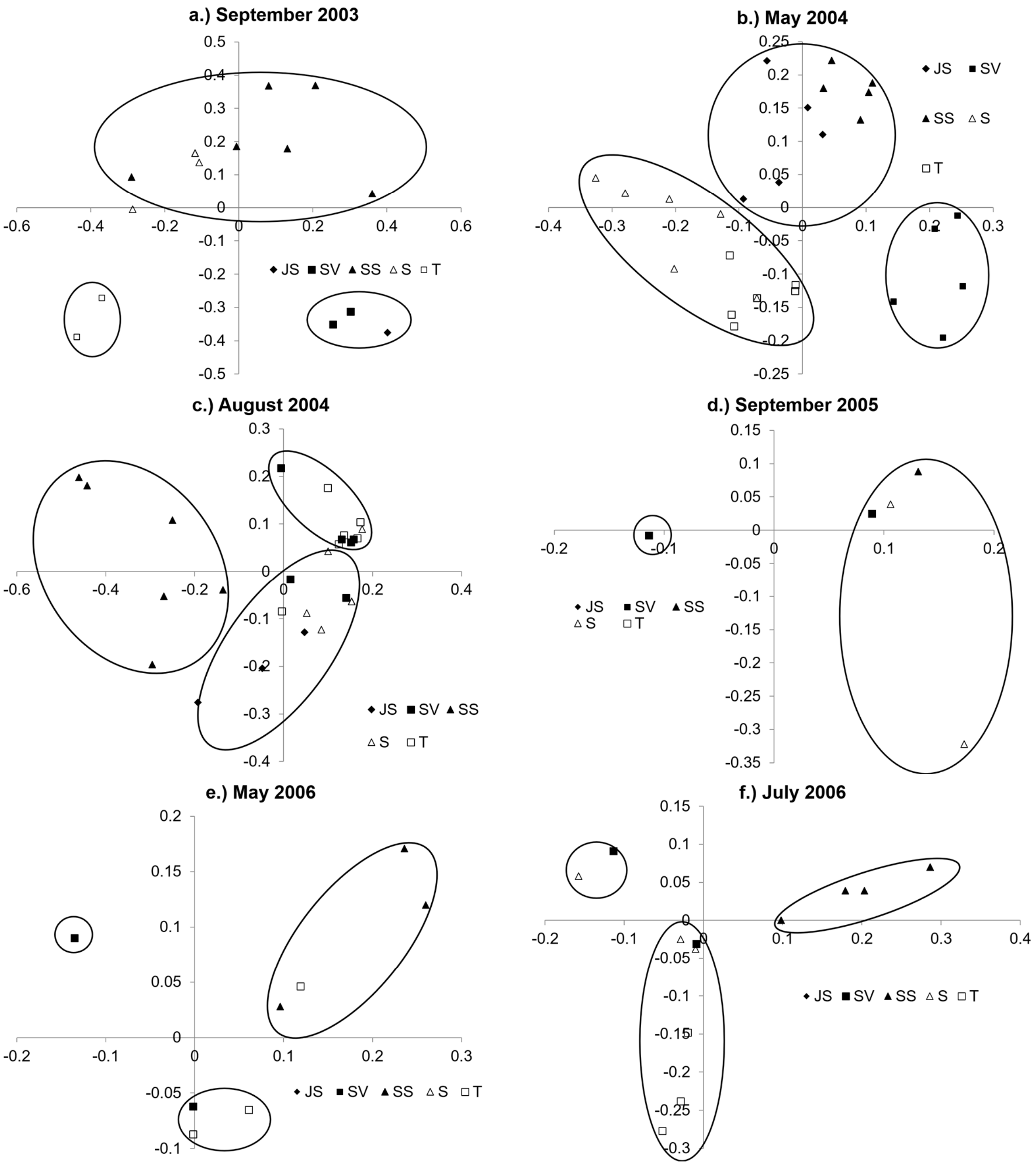

Figure S1: PCA results for dates a.) September 2003, b.) May 2004, c.) August 2004, d.) September 2005, e.) May 2006, and f.) July 2006. Circles denote significance ($p<0.001$) for clustering. Variance per axis for each plot: a.) Axis 1 21.5%, Axis 2 42.2%; b.) Axis 1 23.5%, Axis 2 40.3%; c.) Axis 1 28.6%, Axis 2 42.7%, d.) Axis 1 54.2%, Axis 2 78.7%; e.) Axis 1 39.8%, Axis 2 63.1%; and f.) Axis 1 36.9%, Axis 2 60.1%.

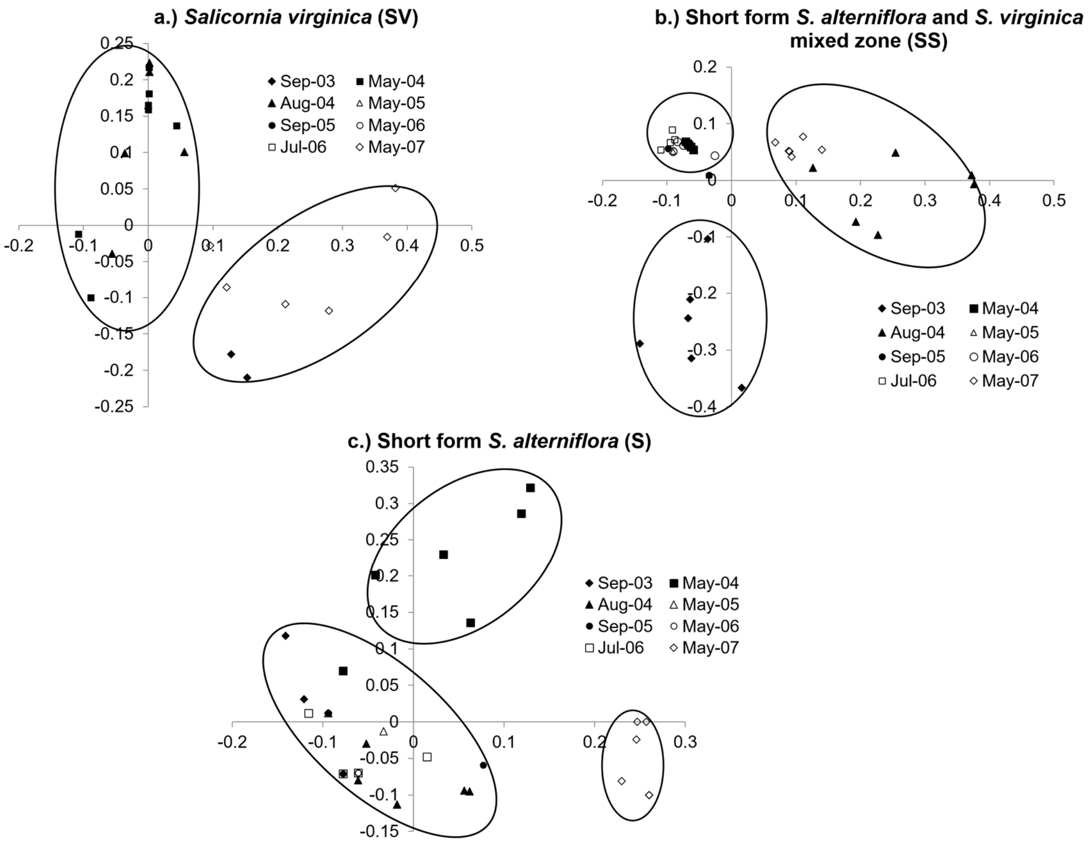

Figure S2: PCA results of a.) *Salicornia virginica*, b.) mid-marsh mixed zone of co-occurring short form *S. alterniflora* and *S. virginica*, and c.) low-marsh short form *S. alterniflora* for all dates. Circles denote significance ($p<0.001$) for clustering. Variance per axis for each plot: a.) Axis 1 25.5%, Axis 2 46.5%; b.) Axis 1 20.6%, Axis 2 37.1%; c.) Axis 1 28.0%, Axis 2 48.6%

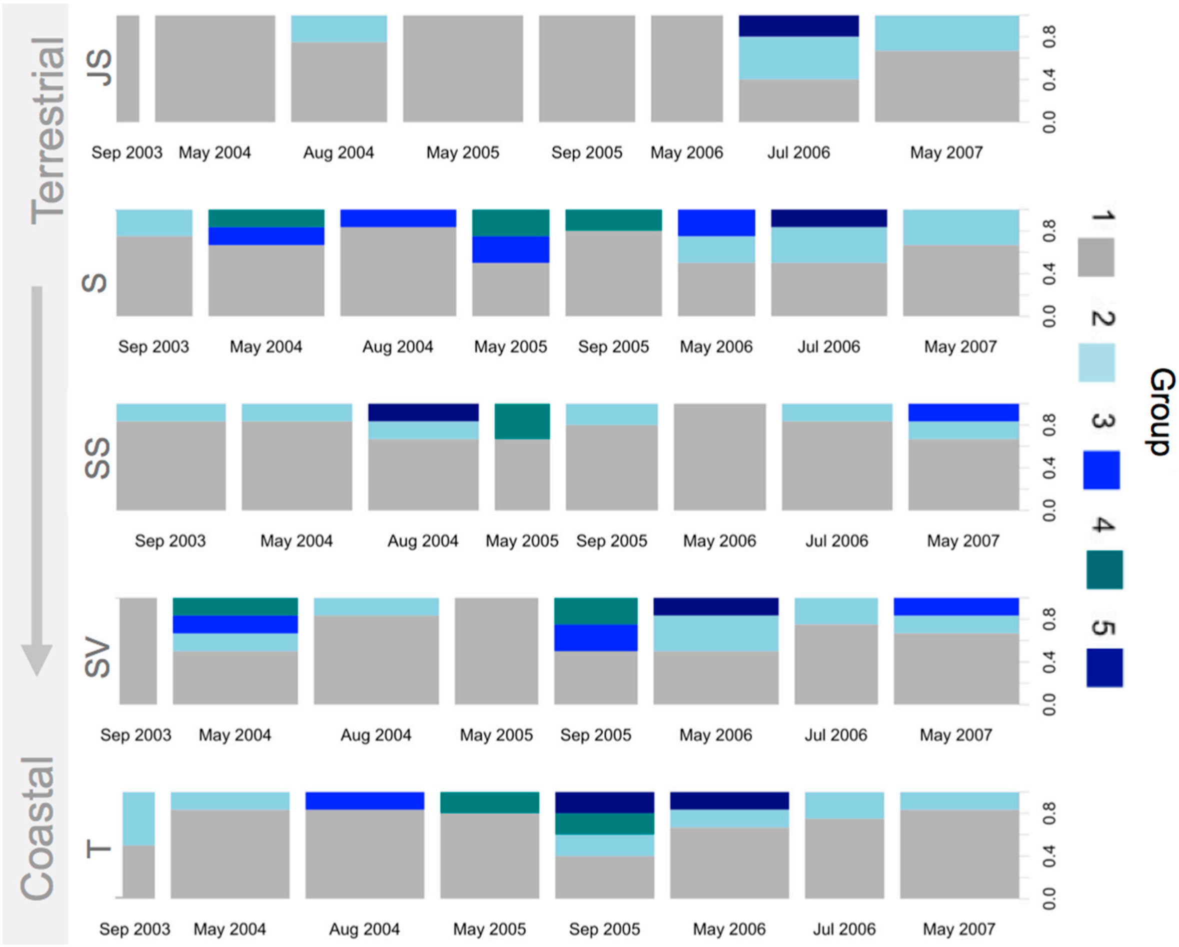

Figure S3. Variation in assemblage groups over time by vegetation zone (JS = high marsh *Juncus roemerianus; S*= low marsh short form *Spartina alterniflora; SV* = high marsh *Salicornia virginica; SS*= mid-marsh mixed zone of co-occurring *Salicornia virginica* and short form *Spartina alterniflora; T* = tall form *Spartina alterniflora*). The width of each block is proportional to the number of core samples with successful DNA isolation and subsequent DGGE gel analysis.

Table S1: Number of samples used per zone for each date in DGGE analysis.

Date / Zone	September 2003	May 2004	August 2004	May 2005	September 2005	May 2006	July 2006	May 2007
**JS**	1	6	4	5	4	3	5	6
**SV**	2	6	6	4	4	6	3	6
**SS**	6	6	6	3	5	5	6	6
**S**	4	6	6	4	5	4	6	6
**T**	2	6	6	5	5	6	3	6
**Total**	15	30	28	21	23	24	25	30

Table S2: Ultimate Band Number (UBN) per diazotroph assemblage group identified through ordination. Bolded numbers in Group 1 relate to sequences obtained and displayed in the neighbor-joining phylogenetic tree in Figure 5.

**Group**	**1**		**2**	**3**	**4**		**5**	
**UBN**	5	**26**	5	5	4	24	4	29
	6	**27**	8	8	5	25	6	31
	8	28	10	9	6	26	8	34
	9	29	12	10	8	27	9	36
	10	30	13	13	9	28	11	38
	**11**	31	14	22	10	29	12	40
	**12**	32	20	30	11	30	13	41
	**13**	34	23	32	12	31	14	42
	15	35	25	33	13	32	15	
	16	36	30	36	14	33	16	
	17	37	31	38	15	34	17	
	18	39	34	40	16	35	18	
	**19**	41	37	41	17	36	20	
	**20**		39		18	37	22	
	21		41		19	43	23	
	**22**		43		20		24	
	**23**		44		21		26	
	24		45		22		27	
	25				23		28	

Table S3: Acetylene reduction assay results. Values are averages of maximum rates of acetylene reduced (nmol/h/mL) based on six replicates per zone for each sampling date. (Avg – Average, SD – standard deviation)

Zone/Date		JS	SV	SS	S	T
**September 2003**	Avg	0	0.16	0.65	0.19	0
	SD	0	0.16	0.09	0.15	0
**May 2004**	Avg	0	0	0.18	0.04	0.15
	SD	0	0	0.28	0.09	0.02
**August 2004**	Avg	0	0.81	0.11	0.18	0.13
	SD	0	0.31	0.09	0.11	0.02
**May 2005**	Avg	0	18.06	33.57	0.43	0.28
	SD	0	11.61	75.07	0.42	0.10
**September 2005**	Avg	0.06	2.17	0.39	1.42	0.07
	SD	0.05	2.29	0.58	1.40	0.04
**May 2006**	Avg	0.69	6.78	3.32	1.30	0.03
	SD	0.88	9.99	4.72	3.05	0.01
**July 2006**	Avg	0.09	0.09	0.08	0.09	0.10
	SD	0.06	0.04	0.50	0.02	0.04
**May 2007**	Avg	0.41	1.15	1.31	1.54	0.17
	SD	0.46	1.99	1.91	1.41	0.04
